# National implementation of multi‐parametric magnetic resonance imaging for prostate cancer detection – recommendations from a UK consensus meeting

**DOI:** 10.1111/bju.14361

**Published:** 2018-06-05

**Authors:** Mrishta Brizmohun Appayya, Jim Adshead, Hashim U. Ahmed, Clare Allen, Alan Bainbridge, Tristan Barrett, Francesco Giganti, John Graham, Phil Haslam, Edward W. Johnston, Christof Kastner, Alexander P.S. Kirkham, Alexandra Lipton, Alan McNeill, Larissa Moniz, Caroline M. Moore, Ghulam Nabi, Anwar R. Padhani, Chris Parker, Amit Patel, Jacqueline Pursey, Jonathan Richenberg, John Staffurth, Jan van der Meulen, Darren Walls, Shonit Punwani

**Affiliations:** ^1^ Centre for Medical Imaging University College London Hospitals NHS Foundation Trust University College London London UK; ^2^ Department of Urology Hertfordshire and Bedfordshire Urological Cancer Centre Lister Hospital Stevenage Hertfordshire UK; ^3^ Division of Surgery and Interventional Science Faculty of Medical Sciences University College London Hospitals NHS Foundation Trust University College London London UK; ^4^ Division of Surgery Department of Surgery and Cancer Imperial College London and Imperial Urology Imperial College Healthcare NHS Trust London UK; ^5^ Department of Radiology University College London Hospitals NHS Foundation Trust University College London London UK; ^6^ Department of Medical Physics University College London Hospitals NHS Foundation Trust University College London London UK; ^7^ Department of Radiology Addenbrooke's Hospital and University of Cambridge Cambridge UK; ^8^ School of Health and Related Research University of Sheffield Sheffield UK; ^9^ Department of Radiology Freeman Hospital Newcastle upon Tyne UK; ^10^ Department of Urology Addenbrooke's Hospital and University of Cambridge Cambridge UK; ^11^ The Society and College of Radiographers London UK; ^12^ Department of Urology NHS Lothian Western General Hospital Edinburgh UK; ^13^ Prostate Cancer UK London UK; ^14^ Department of Urology University College London Hospitals NHS Foundation Trust University College London London UK; ^15^ Division of Cancer Research Ninewells Hospital Dundee UK; ^16^ Paul Strickland Scanner Centre Mount Vernon Hospital Northwood Middlesex UK; ^17^ Department of Academic Urology Royal Marsden Hospital Sutton Surrey UK; ^18^ Department of Radiology Lister Hospital Stevenage Hertfordshire UK; ^19^ Department of Radiology Greater Glasgow and Clyde NHS Trust Glasgow UK; ^20^ Department of Radiology Royal Sussex County Hospital Brighton and Brighton and Sussex Medical School Brighton Sussex UK; ^21^ Division of Cancer and Genetics School of Medicine Cardiff University Cardiff UK; ^22^ London School of Hygiene and Tropical Medicine London UK; ^23^ Division of Nuclear Medicine University College London Hospitals NHS Foundation Trust University College London London UK

**Keywords:** consensus methods, multi‐parametric MRI, prostate cancer, recommendations

## Abstract

**Objectives:**

To identify areas of agreement and disagreement in the implementation of multi‐parametric magnetic resonance imaging (mpMRI) of the prostate in the diagnostic pathway.

**Materials and Methods:**

Fifteen UK experts in prostate mpMRI and/or prostate cancer management across the UK (involving nine NHS centres to provide for geographical spread) participated in a consensus meeting following the Research and Development Corporation and University of California‐Los Angeles (UCLA‐RAND) Appropriateness Method, and were moderated by an independent chair. The experts considered 354 items pertaining to who can request an mpMRI, prostate mpMRI protocol, reporting guidelines, training, quality assurance (QA) and patient management based on mpMRI levels of suspicion for cancer. Each item was rated for agreement on a 9‐point scale. A panel median score of ≥7 constituted ‘agreement’ for an item; for an item to reach ‘consensus’, a panel majority scoring was required.

**Results:**

Consensus was reached on 59% of items (208/354); these were used to provide recommendations for the implementation of prostate mpMRI in the UK. Key findings include prostate mpMRI requests should be made in consultation with the urological team; mpMRI scanners should undergo QA checks to guarantee consistently high diagnostic quality scans; scans should only be reported by trained and experienced radiologists to ensure that men with unsuspicious prostate mpMRI might consider avoiding an immediate biopsy.

**Conclusions:**

Our consensus statements demonstrate a set of criteria that are required for the practical dissemination of consistently high‐quality prostate mpMRI as a diagnostic test before biopsy in men at risk.

## Introduction

The prostate diagnostic pathway is currently based on carrying out a TRUS‐guided biopsy in men with an elevated serum PSA. TRUS‐guided biopsy involves taking 10–12 needle core biopsies from the prostate without prior knowledge as to whether the man has cancer, and if he does, where the cancer resides. This leads to over‐diagnosis of clinically insignificant cancers and missing cancers that are clinically significant, as well as the harms of deploying needles through the rectum.

Multi‐parametric MRI (mpMRI) of the prostate could transform the prostate cancer clinical pathway 1, 2, 3, 4, 5, 6, 7. A recently published prospective multi‐centre study (PROstate MRI Imaging Study [PROMIS]) comparing the diagnostic accuracy of mpMRI and TRUS biopsy demonstrated that mpMRI outperforms TRUS biopsy as a diagnostic tool for the detection of clinically significant cancer. Further, PROMIS showed that a quarter of men at risk could avoid immediate biopsy as a result of unsuspicious mpMRI 8.

However, not all UK institutions have the ability to deliver the benefits of mpMRI to the extent reported within the PROMIS 8. At present, from a Freedom of Information dataset acquired by Prostate Cancer UK, only 50% of centres across UK offer mpMRI to the standard reported within the PROMIS 9.This is because firstly, mpMRI scan quality is variable across centres (of note, ~50% of scanners would need replacement over the next 5 years 9). Secondly, sufficiently experienced radiologists are required to interpret these complex scans. Lastly, there is a lack of detailed guidance for clinicians in how to use mpMRI reports in making decisions in clinic. Sub‐optimal performance in any part of the pathway reduces the potential benefits of introducing mpMRI before first prostate biopsy, risking under‐detection of clinically significant cancers if biopsies are avoided or over‐calling of scans, preventing men from benefiting from mpMRI's triage characteristics of avoiding a biopsy.

To address these challenges, a formal consensus process to determine areas of agreement and disagreement within a panel of UK experts in the field of prostate cancer and/or mpMRI was organised. The aims were to define criteria for requesting, performing and reporting mpMRI scans, addressing quality assurance (QA) of mpMRI, establishing the requirements for mpMRI training, and guiding patient management using mpMRI.

## Materials and Methods

### Design, Setting and Participants

A modified Research and Development Corporation and University of California‐Los Angeles (RAND‐UCLA) Appropriateness Method (RAM) was followed 10. A questionnaire containing 376 items was constructed by six core panelists (H.A., C.A., A.B., A.K., C.M., S.P.) and revisions were made following consultation with other members. The items were identified based on differences in practice across the UK and abroad without duplicating the aims of previous consensus processes. The questionnaire was divided into six sections: (i) Who can request prostate mpMRI, (ii) Prostate mpMRI acquisition protocol updates, (iii) Prostate mpMRI reporting, (iv) QA/quality control (QC) of prostate mpMRI diagnostic process, (v) Management of patients based on prostate mpMRI reports, and (vi) Training in prostate mpMRI.

The diagnostic role of mpMRI in a pre‐biopsy setting in men at risk of prostate cancer was considered and the uses of mpMRI for active surveillance of low‐risk disease or post‐therapy follow‐up were not addressed.

Panelists were selected due to their peer‐reviewed publications and expertise in prostate mpMRI and/or prostate cancer management whilst ensuring a geographical spread. The questionnaire was sent to 21 experts (eight radiologists, seven urologists, two oncologists, three radiographers, and a physicist). Eighteen participated in round 1 and fifteen attended the meeting. An independent non‐scoring moderator with significant experience in leading consensus meetings acted as chair.

#### Round 1: Individual Questionnaire Completion

Panelists were asked to rate their agreement with questionnaire statements for which they considered they had sufficient expertise on a 9‐point scale (ranging from 1 ‘strongly disagree’ to 9 ‘strongly agree’). If they lacked expertise for a particular item, they scored ‘0’ to indicate that they were non‐scoring experts for that item.

#### Round 2: Face‐to‐Face Meeting Discussion

Fifteen attending panel members were shown the first‐round score distribution for each questionnaire statement. After each statement discussion, the panelists rescored the item. Items scored by at least eight panel members were included in the results. Nine consensus statements were added, 23 removed and 39 statements reworded for clarity. Eight items responded to by less than eight panelists were excluded reducing the number of consensus statements to 354.

### Interpretation of the Results

We considered that there was ‘agreement’ with an individual statement for a panel median score of ≥7 and ‘disagreement’ for a panel median score of ≤3. A score between 4 and −6 reflected ‘uncertainty’. Consideration for a particular item to reach ‘consensus’ depended on the number of scoring panel members as elaborated in the RAM 10.

## Results

Pre‐meeting consensus was reached in 127 of 376 items (34%). During the meeting, consensus was reached in 208 of 354 items (59%). Table 1 shows the percentage of items reaching consensus for each section of the questionnaire before and after the face‐to‐face meeting. The Appendix S1 includes the detailed results for each questionnaire item.

**Table 1:** Number (%) of items reaching consensus in each section of the questionnaire

Section	Pre‐meeting	Post‐meeting
	*n/N* (%) consensus items	*n/N* (%) consensus items
I. mpMRI requests	6/12	10/12
II. mpMRI acquisition protocol updates	12/41 (29)	25/41 (61)
III. mpMRI clinical reporting	43/141 (30)	85/131 (65)
IV. QA/QC of mpMRI	44/100 (44)	47/89 (53)
V. Management of patients	12/56 (21)	24/54 (44)
VI. mpMRI training	10/26 (38)	17/27 (63)
Total	127/376 (34)	208/354 (59)

Statements for which consensus was reached are summarised below. Some statements for which consensus majority was not reached are also discussed while mentioning that this was ‘agreement without consensus’.

### Section I: Who Can Request Prostate mpMRI?

The panel agreed in consensus that mpMRI requests should be made by urologists, uro‐oncologists, and specialist urology nurses. The latter would act mostly as a filter to determine the appropriateness of all incoming requests. In the current healthcare environment, any other clinical team may also be able to request prostate mpMRI provided that there is prior urological consultation, to ensure effective communication of mpMRI results and continuity of care. There was consensus that GPs should not directly request prostate mpMRI and patients should not self‐refer for prostate mpMRI.

It was also unanimously agreed that mpMRI should not be offered to all men *prior to clinical assessment* and that an elevated PSA should be assessed with other clinical factors such as age, family history, DRE findings, PSA kinetics and previous TRUS biopsy to determine referral for a prostate mpMRI examination.

### Section II: Updates on Prostate mpMRI Acquisition Protocol

The panel focused on differences between previous UK recommendations 11 and other international consensus guidance such as Prostate Imaging and Reporting and Data System (PI‐RADS_v1 and _v2) 12, 13. The outcomes are summarised in Table 2, with some elaborated on below.

**Table 2:** Prostate mpMRI acquisition protocol updates

Protocol updates
The minimum and optimal field strengths at which prostate mpMRI should be conducted is 1.5 T and 3 T, respectively. Endorectal coils and rectal catheters for gas voiding do not need to be used routinely. Anti‐peristaltic agents should be incorporated in routine practice (unless contra‐indicated). Axial imaging should be orientated axial to the patient and not to the position of the prostate gland.
T2 sequences should be acquired in all three planes and should be obtained as three separate acquisitions (axial, coronal and sagittal). Single 3D T2 imaging sequence was not adequate to replace the three separate 2D acquisitions. T2 sequences with a large field‐of‐view to cover abdominal nodes are not necessary. A maximum voxel size in‐plane resolution of T2 sequences should be 0.7 mm or better.
The minimum high‐*b* value for diffusion‐weighted sequences should be *b* = 1 400 s/mm^2^ at 1.5 T and *b* = 2 000 s/mm^2^ at 3 T. The maximum voxel size in‐plane resolution of DWI should as far as possible ≤2 mm.
Quantitative pharmacokinetic DCE‐MRI modelling or curve shape parametric evaluations are not necessary. DCE analysis should be performed with visual (qualitative) anatomical evaluation in the early arterial enhancement images of the prostate. The temporal resolution of DCE‐MRI sequences can be up to 15 s for a high spatial resolution and anatomical interpretation of DCE images.

Consensus was reached on orientating axial imaging to the patient and not to the position of the prostate gland. Although the latter is the orientation of choice for the correspondence of MRI scans to prostatectomy specimens particularly used in research, in the setting of men undergoing surveillance with repeat scans to monitor any interval change in lesion size, axial imaging to the patient was considered helpful for improving consistency and reproducibility of scans and lesion measurements albeit this requires validation. Also direct intervention of radiologists during the scan is reduced.

#### T2‐Weighted Imaging (T2W)

The panel endorsed the previous statement 12 that this sequence should be acquired in all three planes (the sagittal plane being useful for pre‐surgical planning and improved visualisation of the bladder neck). In particular, T2W should be obtained as three separate acquisitions (axial, coronal and sagittal, two‐dimensional [2D], fast‐spin echo [FSE], multi‐slice) instead of single 3D acquisition until further research on direct comparison of diagnostic quality and cancer conspicuity of 2D vs 3D T2W for both peripheral zone (PZ) and transition zone (TZ) are available 14, 15. The maximum voxel size in‐plane resolution of T2 sequences should be ≤0.7 mm, in keeping with previous recommendations 11, 12. The use of T2 sequences with a large field‐of‐view to cover abdominal nodes outside the pelvis was questioned and was not considered as an essential requirement, as MRI has a poor performance for detection of nodal disease compared to functional imaging techniques such as choline or prostate‐specific membrane antigen positron emission tomography (PET), especially when there is clinical concern of nodal metastatic spread 16, 17.

#### Diffusion‐Weighted Imaging (DWI) Sequences

The minimum high‐*b* value for diffusion sequences should be *b* = 1 400 s/mm^2^ at 1.5 T and *b* = 2 000 s/mm^2^ at 3 T 11, 12. Although consensus was not reached, the majority supported the preference for a separately acquired high *b*‐sequence over an extrapolated/calculated high *b*‐value images. Further evidence on the comparison between ‘extrapolated’ vs ‘separate’ high‐*b* value image acquisitions for histology‐validated prostate cancer detection would be of value 18. The maximum voxel size in‐plane resolution of DWI should as far as possible be kept at ≤2 mm as per previous UK guidelines 11.

#### Dynamic Contrast‐Enhanced Imaging (DCE)

The panel recognised that DCE‐MRI is an essential component of prostate mpMRI for detection, staging and treatment planning 19, 20. DCE‐MRI acts as a ‘safety net’ or a ‘back‐up’ mpMRI sequence especially when DWI images are degraded, which is not uncommon in routine practice (e.g. due to rectal gas). DCE‐MRI analysis should be performed visually, with anatomical evaluation in the early arterial enhancement images of the prostate. Quantitative pharmacokinetic DCE modelling or curve shape parametric evaluation was deemed unnecessary. This means that the temporal resolution can be up to 15 s between scans to allow for a high spatial resolution and anatomical interpretation of DCE‐MRI images.

### Section III: Standards for Prostate mpMRI Clinical Reports

Table 3 summarises the outcomes of this section.

**Table 3:** Consensus recommendations on clinical mpMRI reports

Recommendations on clinical mpMRI reports
The image quality of the mpMRI be reported. mpMRI should be scored to rule out Gleason score 7 (including 3 + prominent 4), and/or volume ≥0.5 mL, and/or extraprostatic extension/seminal vesicle invasion. The mpMRI scoring system recommended is the ‘Likert‐assessment’ system (both for lesion‐scoring and whole‐gland scoring). Equivocal prostate mpMRI (Likert‐impression 3) should be double‐read if avoiding biopsy is under consideration. Discordant mpMRI scores with biopsy results should be retrospectively double‐read.
The following should be scored on a 1–5 scale for likelihood of involvement: ᴑ Extraprostatic extension. ᴑ Seminal vesicle involvement. ᴑ Bladder neck involvement. ᴑ Neurovascular bundle involvement. ᴑ Rectal wall involvement. ᴑ Bladder wall involvement. ᴑ Peripheral zone (PZ) and Transition zone (TZ) tumour should be measured from any sequence on which it is best seen.
The following quantitative metrics should be included within an mpMRI report: ᴑ Prostate gland volume and tumour size should be measured on T2‐weighted imaging using 3‐diameters × 0.52 (prolate ellipse formula). ᴑ To ensure consistency, tumour should be measured as 3‐diameters or volume estimation by the product of 3 diameters × 0.52. ᴑ For software‐targeted biopsy purposes, tumour should be contoured on the sequence required by targeted biopsy fusion software. ᴑ For targeted biopsy purposes, in a lesion >1 cm, the most suspicious area/spot for significant tumour, (i.e. the ‘hot‐spot’) should be additionally indicated (e.g. by contouring, via arrow‐heads, etc.).

#### Who Can Report Prostate mpMRI?

Given the expertise required to report mpMRI, the panel recommended that only uro‐radiologists or radiologists with a specialist interest in prostate cancer imaging should produce prostate mpMRI reports. They should report at least 100 mpMRI examinations per year with the requirement of an active participation in multi‐disciplinary team (MDT) meetings of at least twice a month. Other specialists (general radiologists, MR radiographers, urologists or uro‐oncologists) should only be able to review/demonstrate prostate mpMRI findings within the scope of their practice (e.g. a urologist would review an MRI prior to performing a targeted biopsy, but the images would already have been formally reported by a radiologist with prostate MRI expertise).

#### Significant Cancer Definition Thresholds for mpMRI Assessment

Acknowledging differences in opinions on the definitions of significant prostate cancer, there was consensus agreement to align with the definitions of clinically significant cancer as described in PI‐RADS_v2 12. Specifically, mpMRI should be scored to rule out Gleason score ≥3+4 and/or volume ≥0.5 mL, and/or extraprostatic extension/seminal vesicle invasion.

#### Prostate mpMRI Clinical Reports

There was consensus on the use of a 5‐point Likert‐impression scale (based on the radiologist's overall opinion and experience without the use of a dominant MRI sequence) to rate likelihood of clinically significant disease in routine reporting, as prospectively validated in the multi‐centre PROMIS study 8. There was no consensus on the routine use of the current ‘lesion‐based only’ assessment PI‐RADS_v2 scoring system or a concurrent use of PI‐RADS_v2 and the subjective Likert assessment in the UK. The panel acknowledged the lack of direct comparisons between subjective Likert assessment and PI‐RADS_v2 scoring, as comparisons to date involved Likert assessment and PI‐RADS_v1 21, 22. The majority of the panel disagreed with current PI‐RADS_v2 reporting recommendations that lesion size should be the *only* factor differentiating between a score 4 and 5 for the likelihood of tumour.

**Figure 1:**
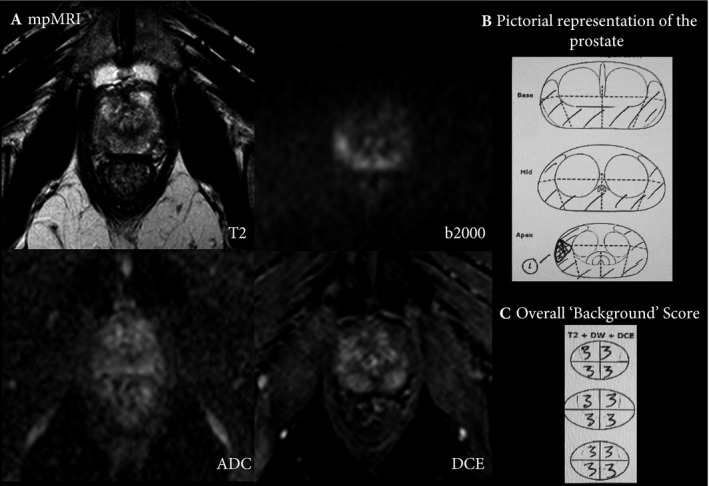
(**A**) Shows the mpMRI of a 62‐year‐old man, with a PSA level of 4.4 ng/mL and a gland volume of 25 mL at the level of the mid‐gland to apex region. On T2W imaging, there is diffuse and patchy low T2 signal and a lower T2 signal at the right lateral gland, with an equivocal high signal focus on diffusion high *b* value at 9 o'clock and corresponding equivocal low ADC signal with bilateral enhancement on DCE. The focal lesion (represented by number 1 in 1. (**B**) was reported with a Likert‐assessment of 3/5. Besides, the remainder of the gland was also assessed with the whole prostate divided into quarters for Likert assessment (**C**). Each quarter was reported as a ‘Likert‐assessment’ 3/5. The background changes scored 3 are represented by the shaded area in **B**. Upon transperineal template mapping biopsy, the prostate was found to harbour adenocarcinoma Gleason 3+4, (40% biopsy core involvement) at the right posterior apex, focal high‐grade prostatic intraepithelial neoplasia at the left posterior apex and Gleason 3+3, at eight different sites within the prostate (10–40% biopsy core involvement).

In addition to lesion‐based assessment, the remainder of the prostate should also be scored on a subjective 5‐point Likert‐assessment scale, to assess the significance of diffuse ‘background’ signal change within the gland, which may potentially mask significant tumour and prompt biopsy (as illustrated in Fig. 1). Of note, whole‐gland assessment is not addressed in PI‐RADS_v2. Prostate and tumour volume should be reported (the sagittal plane to measure the antero‐posterior diameter and height of the gland, and the axial plane for the width of gland were found to be more accurate for gland volume estimation 23; of note, the use of the semi‐ellipsoid formula for lesion volume estimation is practical albeit not yet validated). It is not necessary to report the apparent diffusion coefficient (ADC) values of lesions given the variability between scanners and centres 24, 25. When calculating PSA density, the panel recommended the use of MR‐based volumes over TRUS‐based volume, for greater accuracy 26.

For pictorial prostate diagrams, there was uncertainty over routine use of either a minimum of 12‐sector or extensive 36‐sector PIRADS_v2 diagram. However, the panel all emphasised the benefits of a clear identification and an effective description of all mpMRI visible lesion(s) – these may be either: drawn on any sectored prostate template diagram (either hand‐drawn or computer‐generated); indicated/contoured on the sequence it is best visible in picture archiving communications system; screenshot as key images; saved as annotated images or indicated within the narrative text by sequence and slice numbers.

#### Double‐Reporting

While there is not a need to double‐read all mpMRI, there was agreement in consensus that equivocal prostate mpMRI (Likert‐impression 3) should be double‐read, if avoiding biopsy is under consideration. Also, discordant mpMRI scores with biopsy results should be retrospectively re‐read by a different radiologist. Any uro‐radiologist or radiologist with a specialist interest in prostate mpMRI imaging meeting the minimum requirements for independent reporting would be deemed appropriate to double‐read the scans.

Table 4 shows areas lacking consensus in the mpMRI reporting section.

**Table 4:** Shows areas lacking consensus in clinical mpMRI reporting

Areas lacking consensus in clinical mpMRI reporting
• PI‐RADS_v2 scoring system may be used during training/gaining experience before switching to the use of subjective Likert‐impression once experienced.
• The narrative report should refer to the sectors as named in the PI‐RADS_v2 pictorial report used: e.g. sectors named PZpl (postero‐lateral PZ), PZpm (posteromedial PZ), TZp (posterior TZ), TZa (anterior TZ), etc.).
• In the pictorial report, the prostate diagram should be represented in all three planes.
• mpMRI suspicious lesions contouring should be performed only when targeted biopsy or focal treatment is planned.
• Tumour volume should be calculated by summation of contoured areas on each slice of the tumour/software rendering.
• Transition zone (TZ) tumour should be measured from T2 only (as in PI‐RADS_v2).

### Section IV: QA/QC of Prostate mpMRI

Setting national quality standards to perform prostate mpMRI was envisaged in this section, to parallel breast imaging, where national quality standards are established to ensure safe, reliable, and accurate imaging services at accredited facilities.

However, as prostate cancer is not part of a national screening programme, consensus was not achieved to define stringent QA standards equivalent to breast imaging. This would ultimately be within the remit and expertise of accreditation bodies to be implemented in the future. Nevertheless, some areas of the panel discussion covering site‐specific, scanner‐specific, image‐specific and radiologist‐specific QA aspects are highlighted below.

Although consensus was not reached, a majority of 67% supported accreditation for sites performing prostate mpMRI, which would be administered by a national body. An accredited centre should be able to perform T2W, DWI and DCE‐MRI to the latest national guidelines and perform or refer patients for biopsy, MDT‐meeting discussions, and treatment.

Moreover, it was agreed in consensus that every scanner should undergo regular QA/QC procedures in order to perform prostate mpMRI. This is already a routine scanner QA requirement within NHS institutions. Detailed guidance is available on the American College of Radiology (ACR)/Association of American Physicists in Medicine (AAPM) website 27.

For diagnostic image‐quality assessment, qualitative and quantitative assessments were discussed. Whilst the usefulness of quantitative image assessment was unclear, qualitative assessment through visual image assessment by a radiologist analysing the images (e.g. looking for artefacts to prompt correction, assessing lesion conspicuity etc.) was recognised as adequate to determine diagnostic acceptability.

For radiologists to maintain reporting performance over time, the panel supported the use of combined self‐performance tests, external performance assessments, and institution‐based audits. The development of any online performance assessment tools could feature non histology‐validated MRI cases to compare the radiologist's performance to experts and/or use histology‐validated cases to evaluate the radiologist's sensitivity, specificity, false negatives/positives, and accuracy for significant cancer.

It was recognised that these performance characteristics would not only reflect the expertise of the biopsy operator but also the reporting radiologist. These assessments would help identify under‐performing radiologists to motivate self‐improvement, such as increasing the number of mpMRI reporting backed by continuous feedback from experts or peer‐reviewers, second‐reading by an experienced radiologist for a set period, increasing mpMRI to pathology correlations, e.g. over the next 6–12 months, before any re‐evaluation.

### Section V: Management of Patients After Prostate mpMRI Results

Whilst not formally considering all clinical factors as items for consensus voting, it was clear that pre‐biopsy mpMRI scoring should not be the only factor guiding decisions about whether to biopsy. Other factors such as age, family history, use of 5α‐reductase inhibitors, comorbidities, total PSA, PSA kinetics, PSA density, urine dipstick tests (to exclude infection), prior biopsy results, and patient preference, might be considered in conjunction.

When pre‐biopsy mpMRI is scored 1–2 (not suspicious for clinically significant cancer) and the PSA density is below an agreed threshold, it was agreed in consensus that the patient can be discharged to the GP with PSA follow‐up, i.e. no immediate biopsy is required. However, where PSA density is above the agreed threshold, then biopsy should be discussed with the patient as part of a shared decision‐making process discussing risks of disease being present (~0–24% of negative mpMRI harbour significant cancer depending on the definition used 2, 8, 28, 29, 30, 31, 32, 33, 34 and the risks related to the biopsy procedure. The recommended biopsy technique for men who on clinical grounds, are advised or choose to have a biopsy despite a unsuspicious mpMRI is by any transperineal systematic biopsy (10/12, 83% panelists) due to no visible MR target; systematic TRUS biopsy would only increase the detection rate by 1–2% 31, 32 and was not deemed worthwhile in the presence of unsuspicious mpMRI.

For equivocal mpMRI impressions (Likert‐assessment 3), a biopsy is recommended when the PSA density is above an agreed threshold (unanimous consensus). There was agreement but not in consensus that young patients (seven of 12 panelists) and with positive family history (eight of 12 panelists) could also undergo biopsy if the mpMRI was scored 3. It was agreed but not in consensus that biopsy options in equivocal mpMRI should include MR‐guided biopsy (visually estimated, image fusion or in‐bore; eight of 12 panelists), transperineal systematic biopsy (seven of 12 panelists) or combined targeted and systematic sampling (seven of 12 panelists) of the gland. Emerging evidence shows no statistical difference between the use of combined targeted and systematic biopsy vs each biopsy technique alone for significant cancer detection in equivocal lesions 35.

Immediate biopsy is recommended for suspicious pre‐biopsy mpMRI (Likert‐assessment 4–5); Suspicious mpMRI followed by negative targeted biopsy should be discussed as part of an MDT meeting for collective management decision, where the possibility of a missed targeted biopsy, or of a false‐positive mpMRI report would both be considered. Table 5 summarises this section's discussion.

**Table 5:** Recommendations for incorporation of mpMRI scores in patient's management

Recommendations for incorporation of mpMRI scores in patient's management
mpMRI scores 1–2
• No immediate biopsy is recommended.
• Biopsy can be considered as part of a shared decision process with the patient if • PSA density is elevated or clinical concerns persist.
mpMRI score 3
• Immediate biopsy if PSA density is elevated.
mpMRI scores 4–5
• Immediate biopsy.
mpMRI scores 4–5 and targeted biopsy is negative
• Discuss in MDT meeting.

### Section VI: Training in Prostate mpMRI Reporting

It was unanimously agreed that prostate mpMRI reporting cannot be self‐taught. Before commencing independent mpMRI reporting, radiologists should undertake a combination of core theoretical prostate mpMRI course, hands‐on practice at workstations with supervised reporting, and should also participate in MDT meetings or attend MDT‐type workshops where patient‐based clinical scenarios are discussed. Training should be dispensed and certified by national bodies such as the Royal College of Radiologists. Table 6 highlights the areas of agreement of the panel within this section.

**Table 6:** Summarises the recommendations in the training section

Agreement in consensus
There should be a competency examination in prostate mpMRI prior to starting independent reporting. Attendance on a training course should be made mandatory prior to starting independent reporting. There should be evidence of self‐directed learning. Prostate mpMRI training course for non‐reporters should differ from the reporters’ course and adapted to their specialty field. There should be a national accreditation for prostate mpMRI reporting. Certified, standardised training for prostate mpMRI should be provided by a national body.
Prior to commencing independent mpMRI reporting, reporters should attend a combination of: ᴑ A core theoretical mpMRI course. ᴑ Hands‐on practice at workstations. ᴑ Supervised reporting. ᴑ MDT‐type workshops aimed at discussing patient‐based clinical scenarios. Hands‐on training may be given by centres carrying out a minimum number of ≥250 cases/year.

**Figure 2:**
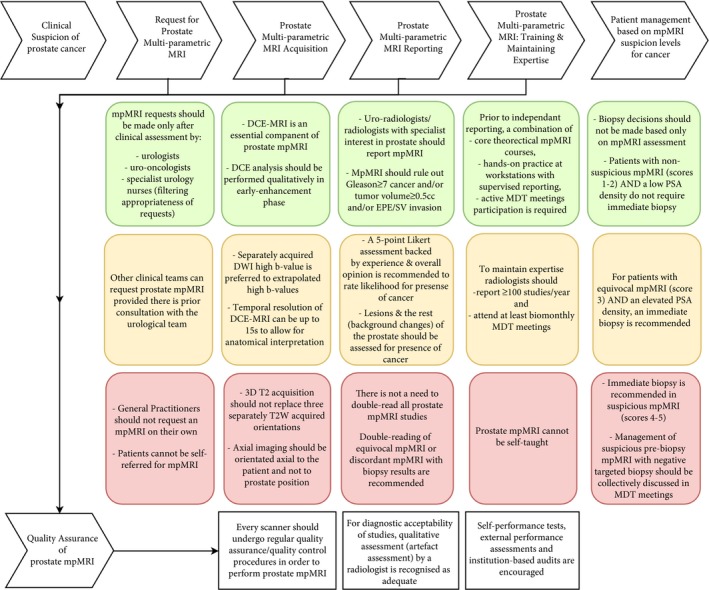
Summarises the key recommendations across the early prostate cancer diagnosis pathway to deliver consistently high‐quality mpMRI studies.

Figure 2 summarises key recommendations from this process.

## Discussion

The present paper reports formal UK recommendations from a RAM consensus process to guide the practice of pre‐biopsy prostate mpMRI in expert and non‐expert centres. Recommendations have been made to ensure consistently high‐quality mpMRI scans and improve standards in reporting to offer better guidance in management decisions.

### Clinical Implications

There are a number of key statements that we believe will be of significant impact in the UK prostate cancer diagnostic pathway.

First, we have laid down the minimum conditions for a prostate mpMRI. This includes the added value of DCE‐MRI, which has been lately questioned 36, 37, 38, 39, 40, 41. A recent UK audit revealed that 24% of NHS centres do not conduct prostate MRI with DCE 9; it was found that the main reason for non‐compliance with the UK and PI‐RADS guidelines 11, 12 was due to capacity problems. However, the panel still recommends the use of DCE as integral to prostate MRI, i.e. which evolves from bi‐parametric (T2W and DWI) to mpMRI, with the addition of DCE‐MRI. The benefits of DCE‐MRI include the fact that it acts as a back‐up to overcome technical failures from DWI and artefacts (such as susceptibility artefact from rectal gas and distortion from hip replacement), which hinder diagnostic accuracy 19, 42, 43, 44, 45. Besides, DCE‐MRI is helpful for the less experienced radiologists 46 and also beneficial in differentiating the anterior fibromuscular stroma from anterior tumours 47. DCE‐MRI can also act as a ‘safety’ sequence in patients where diffusion images are significantly distorted by susceptibility artefact from air within the rectum or total hip replacements. Furthermore, DCE also improves the confidence regarding identified lesions particularly for those of an indeterminate nature 19, 48, 49.

Second, we also questioned the widespread use of PI‐RADS_v2 scoring system and recommended the use of a subjective 5‐point Likert‐assessment of mpMRI scans in the UK pending higher‐level validation and further evolution of the PI‐RADS scoring system (noting that PI‐RADS_v3 is currently under development). Although PI‐RADS_v2 promotes an objective lesion‐based scoring approach 12, it does not include routine assessment of the whole prostate, e.g. the significance of diffuse ‘background’ signal change within the whole gland is often not addressed 50. Even though the dominance of a sequence in PZ/TZ lesion evaluation was acknowledged, tumours exhibiting strong cancer suspicion on the non‐dominant sequence (e.g. T2 in the PZ or ADC in the TZ) could be missed with PI‐RADS_v2. Also, the latter does not fit in other areas, i.e. central zone, anterior stroma and zonal interface where zonal origin unclear. These are areas where a ‘Likert‐impression’ score can be more useful. Nevertheless, radiologists can use the descriptive scoring characteristics as elaborated in PI‐RADS_v2 to guide their opinions and supplement them with their own experience, as well as features outside of PI‐RADS_v2 criteria to form an overall subjective Likert‐impression. The panel also acknowledged that mpMRI descriptive features in the PIRADS_v2 guidelines are particularly useful for the less experienced and for research. Furthermore, they discussed the need for a histologically‐validated pictorial guide to illustrate subjective Likert‐impressions and this could be delivered through datasets acquired during clinical trials.

Third, in order to maintain quality mpMRI reporting and guarantee accurate and safe prostate mpMRI reports, minimum standards for reporting radiologists were tackled. Although the effect of dedicated training on the accuracy of prostate cancer localisation on mpMRI, the effect of continual feedback on reporting confidence and a ‘learning curve’ effect have been documented, the establishment of a threshold number of prostate mpMRI required during training, to reach independent reporting and to maintain expertise are lacking 51, 52, 53, 54. While some may not agree with the concept of quantitative metrics to gauge quality or experience, the majority of the panel agreed that an independent radiologist should report >100 prostate mpMRI scans per year with regular attendance to MDT meetings of at least twice a month. Also, prior to independent reporting, supervised reporting of at least 100 mpMRI studies were deemed appropriate. Moreover, centres carrying out at least 250 cases per year were regarded as best suited to dispense training. These numbers are under the proviso that the scans also meet the minimum quality requirement as per the latest protocol guidelines. Furthermore, it was stressed that general radiologists are not to report prostate mpMRI unless they have a specialist interest in prostate mpMRI, and like uro‐radiolgists are prepared to meet the necessary minimum requirements in terms of training and experience, prior to autonomous reporting.

This expert group initiated the discussion regarding prostate mpMRI‐specific QA, but it was recognised that more specialist technical groups with specific QA expertise are required to set‐up relevant QA requirements across the whole prostate cancer pathway including QA procedures for pathology, surgery, and data collection. QA requirements for breast cancer diagnosis pathway 55 could be used as an exemplar approach.

Last, we considered who should be biopsied based on mpMRI reports. There was consensus that mpMRI report should be used to determine whether a man should be biopsied, capitalising on the high sensitivity and high negative‐predictive values for mpMRI in excluding clinically significant prostate cancer and between one‐quarter and one‐third of men would be given the opportunity to avoid an immediate biopsy. Growing literature on the combined use of PSA density with mpMRI as an additional factor to reduce the false negatives of mpMRI 50, 56, 57, 58, 59, 60, 61 was endorsed by the panel to better select patients for biopsy after non‐suspicious and equivocal mpMRI 35, 57, 62. Whilst various PSA density thresholds have been previously suggested 63, 64, 65, the threshold of 0.15 ng/mL/mL is proving to be useful in the diagnostic setting 57, 58, 59, 66, although individual centres may choose to be more conservative in using lower PSA density threshold (e.g. 0.12 ng/mL/mL) alongside other risk factors in deciding which men can avoid a biopsy until more robust evidence is available.

### Research Implications

Some areas did not reach consensus, due to conflicting results or lack of data in the literature to guide discussions. Areas of further research include: lesion detection/conspicuity comparisons from dedicated vs extrapolated/computed long *b*‐values at 3 T and 1.5 T without endorectal coils; quantitative image quality assessments; specific MR‐prostate phantom development; threshold number of mpMRI studies required during and after prostate mpMRI training and to reach autonomous reporting; long‐term clinical risk of cancer and outcomes of high‐grade prostatic intraepithelial neoplasia (HGPIN), atypical small acinar proliferation (ASAP), atrophy or inflammation upon diagnosis for mpMRI directed management options 67, 68; and combining mpMRI with molecular/genomic biomarkers, risk‐calculators (other than TRUS‐biopsy validated ones) across the prostate cancer pathway for diagnosis.

### Methodological Limitations

Expert group discussions are prone to biases, but latest available evidence was used and an independent chair ensured balanced debates. Even if one or two panelists dominated the discussion, they had only one vote. Besides, some members of the panel scored the items before the meeting but were not present in the face‐to‐face meeting. Whilst a GP representative on our panel would have been beneficial to address the initial questions involving GPs, the contribution to the remainder of the document would be limited. Finally, this process does not aim to reach consensus in areas of disagreement or minimise uncertainties in clinical areas but it has helped to identify areas warranting additional research.

## Conclusions

The promise of mpMRI is reflected by the rapid uptake of this investigation into clinical practice and the growing demand to offer this test across the UK. Our consensus statements demonstrate a set of criteria that are required for the reliable dissemination of prostate mpMRI as a diagnostic test prior to biopsy in men at risk. It is of utmost importance that quality should be maintained across the whole prostate pathway in all healthcare settings for prostate mpMRI to be used as a tool to rule in and rule out clinically significant prostate cancer.

## Participating Members and Author Contributions

Meeting Chair: Jan van der Meulen.

Panelists who agreed to be part of the process: eight radiologists (Clare Allen, Tristan Barrett, Alexander P.S. Kirkham, Phil Haslam, Anwar R. Padhani, Amit Patel, Jonathan Richenberg, Shonit Punwani), seven urologists (Jim Adshead, Hashim U. Ahmed, John Graham, Christof Kastner, Alan McNeill, Caroline M. Moore, Ghulam Nabi), two oncologists (Chris Parker, John Staffurth), three radiographers (Darren Walls, Jacqueline Pursey, Alexandra Lipton) and a physicist (Alan Bainbridge).

Panelists who completed the questionnaire: Clare Allen, Tristan Barrett, Alexander P.S. Kirkham, Phil Haslam, Amit Patel, Jonathan Richenberg, Shonit Punwani, Hashim U. Ahmed, Jim Adshead, Christof Kastner, Caroline M. Moore, Ghulam Nabi, Chris Parker, John Staffurth, Darren Walls, Jacqueline Pursey, Alexandra Lipton, Alan Bainbridge.

Panelists who participated in face‐to‐face meeting: Clare Allen, Tristan Barrett, Alexander P.S. Kirkham, Phil Haslam, Amit Patel, Jonathan Richenberg, Shonit Punwani, Hashim U. Ahmed, Christof Kastner, Caroline M. Moore, Chris Parker, John Staffurth, Darren Walls, Jacqueline Pursey, Alan Bainbridge.

Meeting Coordinator: Mrishta Brizmohun Appayya.

Critical revision of the questionnaire and manuscript for important intellectual content: Jim Adshead, Hashim U. Ahmed, John Graham, Christof Kastner, Alan McNeill, Caroline M. Moore, Ghulam Nabi, Chris Parker, John Staffurth; Darren Walls, Jacqueline Pursey, Alexandra Lipton, Alan Bainbridge; Clare Allen, Tristan Barrett, Francesco Giganti, Edward W. Johnston, Alexander P.S. Kirkham, Phil Haslam, Anwar R. Padhani, Amit Patel, Jonathan Richenberg, Jan van der Meulen, Shonit Punwani.

Elaboration of questionnaire: Mrishta Brizmohun Appayya, Hashim U. Ahmed, Clare Allen, Alan Bainbridge, Alexander P.S. Kirkham, Caroline M. Moore, Shonit Punwani.

Study concept and design: Mrishta Brizmohun Appayya, Shonit Punwani.

Analysis and interpretation of data: Mrishta Brizmohun Appayya.

Statistical analysis: Mrishta Brizmohun Appayya.

Obtaining funding: Shonit Punwani.

Administrative, technical, or material support: Edward W. Johnston, Francesco Giganti, Larissa Moniz, Chris Whittle, Karen Stalbow.

Supervision: Shonit Punwani.

## Conflict of Interest

None.

**Abbreviations**
(2(3)D: two (three)‐dimensional
ADC: apparent diffusion coefficient
DCE: dynamic contrast‐enhanced imaging
DWI: diffusion‐weighted imaging
MDT: multi‐disciplinary team
mpMRI: multi‐parametric MRI
PI‐RADS (_v1)(_v2)(_v3): Prostate Imaging and Reporting and Data System (version 1) (version 2) (version 3)
PROMIS: PROstate MRI Imaging Study
PZ: peripheral zone
QA: quality assurance
QC: quality control
RAM: RAND‐UCLA Appropriateness Method
RAND‐UCLA: Research and Development Corporation and University of California‐Los Angeles
T2W: T2‐weighted imaging
TZ: transition zone

## Supporting information


**Appendix S1.** Detailed results for each questionnaire item.
