# Supplementary material for: National implementation of multi‐parametric magnetic resonance imaging for prostate cancer detection – recommendations from a UK consensus meeting
Source: BJU Int. 2018 Jun 5;122(1):13–25. doi: 10.1111/bju.14361 (PMC6334741; doi:10.1111/bju.14361)
Supplement: Supplementary file 1 — Appendix S1. Detailed results for each questionnaire item. [file BJU-122-13-s001.pdf]

			Agreement		Uncertainty		Disagreement		% Score Distribution of panelists			Abstention
			Consensus	No consensus	Consensus	No consensus	Consensus	No consensus	Score range of **1-3**	Score range **4-6**	Score range **7-9**	n out of 15
		**SECTION I - Who can request a prostate mpMRI**										
Item no.	1A.	In the current health environment, mpMRI requests can be made directly by										
1	a.	Patient					x		100			0
2	b.	GP					x		93	7		0
3	c.	Urologist	x								100	0
4	d.	Urologist with a specialist interest in prostate cancer	x								100	0
5	e.	Radiologist		x					7	26	67	0
6	f.	Uro-oncologist	x								100	0
7	g.	Any clinical team without urological input					x		93	7		0
8	h.	Specialist urology nurse	x							7	93	0
	1B.	mpMRI should be offered										
9	a.	To all men					x		100			0
10	b.	Based on PSA only						x	57	22	21	1
11	c.	Based on PSA and other clinical criteria (PSA kinetics, previous TRUS biopsy, family history, DRE…)	x								100	1
12	d.	At the urologist’s discretion	x							20	80	0

	Agreement		Uncertainty		Disagreement		% Score Distribution of panelists			Abstention
	Consensus	No consensus	Consensus	No consensus	Consensus	No consensus	Score range of **1-3**	Score range **4-6**	Score range **7-9**	n out of 15
**SECTION II – mpMRI Acquisition Protocol**										
2. The minimum field strength at which a prostate mpMRI should be conducted is										
13 a. 1.5T	x								100	0
14 b. 3T					x		87	13		0
3. The optimal field strength at which a prostate mpMRI should be conducted is										
15 a. 1.5T				x			47	33	20	0
16 b. 3T	x									0
17 4. An endo-rectal coil should be used routinely.					x		93	7		0
18 5. Anti-peristaltic agents should be use routinely.	x						13	13	74	0
19 6. A flatus tube should be used routinely.					x		87	13		0
20 7. A flatus tube should be used only if there is prominent rectal air.				x			27	46	27	0
21 8. mpMRI protocol should be tailored for detection of tumour within the prostate only.						x	53	7	40	0
22 nodal/bony staging.				x			20	33	47	0
10. DCE (Dynamic Contrast Enhanced Imaging) is an essential component of prostate mpMRI protocol										
23 a. For detection	x							7	93	0
24 b. For staging	x						20		80	0
25 c. For treatment planning eg focal therapy, surgery or radiotherapy planning	x						20		80	0

		Agreement		Uncertainty		Disagreement		% Score Distribution of panelists			Abstention
		Consensus	No consensus	Consensus	No consensus	Consensus	No consensus	Score range of **1-3**	Score range **4-6**	Score range **7-9**	n out of 15
26	11. T2 should be acquired in all three planes.	x							7	93	1
27	12. 3D T2 can be used to replace separate T2 acquisitions for each of the three planes.					x		91	9		4
28	13. A T2 sequence (eg T2 FISP) with a large field of view is required to cover abdominal nodes.					x		92	8		2
	14. Axial acquired images should be										
29	a. True axial to the patient (UK Consensus 2013)	x								100	3
30	b. Axial to the prostate gland (orthogonal to the rectum) (PI-RADS v1, 2012)					x		100			4
31	15. A dedicated long b-sequence should be preferred to an extrapolated/calculated long b image. (Either extrapolated or dedicated long b is recommended by PI-RADS v2; dedicated long b is recommended from past UK Consensus 2013)		x					8	25	67	3
32	16. The calculated long b-value should be used instead of a dedicated long b-sequence only if SNR limitations arise with the latter.				x			18	46	36	4
	17. At 1.5T, the minimum acceptable value for the diffusion high b-sequence is										
33	a. 1000					x		75	17	8	3
34	b. 1200						x	59	33	8	3
35	c. 1400 (PI-RADS v2; UK Consensus 2013)	x							7	93	2
36	d. 1600						x	50	42	8	3

		**Agreement**		**Uncertainty**		**Disagreement**		**% Score Distribution of panelists**			**Abstention**
		Consensus	No consensus	Consensus	No consensus	Consensus	No consensus	Score range of **1-3**	Score range **4-6**	Score range **7-9**	n out of 15
	18. At 3T, the minimum acceptable value for the diffusion high b-sequence is										
37	a. 1400					x		75	17	8	3
38	b. 1600				x			42	58		3
39	c. 2000 (PI-RADS v2; UK Consensus 2013)	x							7	93	2
40	d. 3000						x	67	25	8	3
	19. The maximum voxel size in-plane resolution of T2 sequences should be										
41	a. ≤ 0.5mm (phase resolution PI-RADS v2)				x			17	50	33	3
42	b. ≤ 0.7mm (frequency resolution PI-RADS v2; UK Consensus 2013)	x							7	93	2
43	c. ≤ 1.0 mm						x	55	36	9	4
	20. The maximum voxel size in-plane resolution of DWI sequences should be										
44	a. ≤ 1.5 mm				x			46	36	18	4
45	b. ≤ 2 mm (UK Consensus 2013)	x						8	15	77	2
46	c. ≤ 2.5 mm (PI-RADS v2)		x					27	19	54	4
	21. The temporal resolution of DCE should be										
47	a. ≤ 7s (preferable as per PI-RADS v2)				x			50	33	17	4
48	b. ≤ 10s (recommended by PI-RADS v2)				x			33	25	42	4
49	c. ≤ 15s (minimum recommendations as per PI-RADS v1 2012, and UK Consensus 2013	x							25	75	3

			Agreement		Uncertainty		Disagreement		% Score Distribution of panelists			Abstention
			Consensus	No consensus	Consensus	No consensus	Consensus	No consensus	Score range of **1-3**	Score range **4-6**	Score range **7-9**	n out of 15
	22.	DCE analysis should be performed using								91		
50	a.	Full quantitative pharmacokinetic DCE modelling					x		92	8		3
51	b.	Curve shape parametric evaluation					x		75	8	17	3
52	c.	Visual evaluation of the early arterial (10-20 second) post-contrast images	x								100	3
53	d.	DCE analysis should not be performed.					x		82	9	9	4
		**SECTION III – mpMRI Clinical Reporting**										
	23.	mpMRI reports should be produced by										
54	a.	General Radiologists					x		86	7	7	0
55	b.	Radiologists with a special interest in prostate cancer/Uro-radiologists	x								100	0
56	c.	Urologists					x		100			0
57	d.	Urologists with a special interest in prostate cancer					x		93		7	0
58	e.	Oncologists					x		100			0
59	f.	Oncologists with a special interest in prostate cancer/Uro-oncologists					x		93	7		0
60	g.	Radiographers					x		100			0
61	h.	Radiographers with a special interest in prostate cancer					x		100			0

		Agreement		Uncertainty		Disagreement		% Score Distribution of panelists			Abstention
		Consensus	No consensus	Consensus	No consensus	Consensus	No consensus	Score range of **1-3**	Score range **4-6**	Score range **7-9**	n out of 15
	24. In the current healthcare system, the following should be able to review a prostate mpMRI within their scope of practice										
62	a. General Radiologists	x							7	93	0
63	b. Uro-radiologists	x								100	0
64	c. Uro-oncologists	x								100	1
65	d. Urologists	x								100	0
66	e. MR radiographers	x							13	87	0
67	f. Therapy radiographers					x		80	7	13	0
68	25. It is necessary for GPs to understand prostate mpMRI reports.					x		93		7	0
	should report per year should be										
69	a. At least 50						x	62	15	23	2
70	b. At least 100	x						16		84	2
71	c. At least 150		x					38	8	54	2
72	d. At least 200						x	69		31	2
	radiologist should actively participate per year is										
73	a. At least 42/yr (once a week – allowing for annual leave)					x		100			2
74	b. At least 24/yr (twice a month)	x							7	93	2
75	c. At least 12/yr (once a month)						x	70	15	15	2

#	Item	Agreement: Consensus	Agreement: No consensus	Uncertainty: Consensus	Uncertainty: No consensus	Disagreement: Consensus	Disagreement: No consensus	% Score Distribution of panelists: Score range of **1-3**	Score range **4-6**	Score range **7-9**	Abstention: n out of 15
	28. mpMRI should be scored to rule out cancer of any volume containing										
76	a. Gleason ≥3+3					x		100			3
77	b. Gleason ≥3+4				x			42	25	33	3
78	c. Gleason ≥4+3	x							8	92	3
79	d. Gleason ≥4+4	x								100	3
	following maximum diameter										
80	a. > 2mm					x		92	8		3
81	b. > 5mm						x	58	25	17	3
82	c. > 7mm		x					17	25	58	3
83	d. > 10mm	x							8	92	3
84	e. No particular threshold is required as long as the lesion is measurable										
	following volume										
85	a. Any volume					x		100			3
86	b. > 0.1cc (4 mm diameter lesion)					x		77	58		2
87	c. > 0.2cc (7mm diameter lesion)				x			31	23	46	2
88	d. > 0.5cc (10mm diameter lesion)	x								100	2
89	e. > 1.0cc (14mm diameter lesion)	x								100	2

		Agreement		Uncertainty		Disagreement		% Score Distribution of panelists			Abstention
		Consensus	No consensus	Consensus	No consensus	Consensus	No consensus	Score range of **1-3**	Score range **4-6**	Score range **7-9**	n out of 15
	histological grade and volume combination										
90	a. Gleason 3+3, > 0.5cc				x				58	42	3
91	b. Gleason 3+3, > 1.0 cc	x						69	8	84	3
92	c. Gleason 3+4, of any volume						x	50	25	25	3
93	d. Gleason 3+4, > 0.2cc				x			16	42	42	3
94	e. Gleason 3+4, > 0.5cc	x						8	8	84	3
95	f. Gleason ≥4+3, of any volume		x					17	17	66	3
96	g. Gleason ≥4+3, > 0.2cc	x						8	8	84	3
97	h. PI-RADS v2 definition	x							16	84	3
	defined as per										
98	a. Gleason score only					x		92	8		3
99	b. Maximum cancer core length (MCCL) only					x		92	8		2
100	c. A combination of Gleason score and MCCL.	x						8	8	84	2
101	d. Either Gleason score or MCCL.						x	69	8	23	2
	33. The following Prostate mpMRI scoring systems may be used for routine reporting in the UK										
102	a. Subjective (Likert-scoring) system	x								100	2
103	b. PI-RADS v2 scoring system		x					25	17	58	3
104	c. PI-RADS v1 scoring system					x		100			2
105	34. Subjective (Likert) and PI-RADS scoring systems should be used concurrently.					x		92		8	3
106	the use of subjective Likert scoring once experienced.						x	54	10	36	4

		Agreement		Uncertainty		Disagreement		% Score Distribution of panelists			Abstention
		Consensus	No consensus	Consensus	No consensus	Consensus	No consensus	Score range of **1-3**	Score range **4-6**	Score range **7-9**	n out of 15
107	36. It is important to distinguish between scores 1 and 2 within the negative areas of the prostate gland on mpMRI.					x		91	9		4
108	37. Size criteria should be the only factor to differentiate between mpMRI Likert score 4 and 5. (in PI-RADSv2, a lesion is scored 5 only if it’s size is > 1.5cm)					x		100			5
109	38. The background changes within the prostate gland should be scored.	x						27		73	4
110	39. The image quality of the mpMRI be reported.	x								100	7
111	e.g sectors named PZpl (postero-lateral PZ), PZpm (posteromedial PZ), TZp (posterior TZ), TZa (anterior TZ), etc) as illustrated below.		x					17	17	66	3
112	41. The narrative report should refer to sectors as named in the pictorial report used in PI-RADS v1: e.g 12a, 3a, 4p					x		92		8	3
	42. Scores for individual sequences should be included in the final report when using the following scoring systems										
113	a. Subjective (Likert) scoring					x					3
114	b. PI-RADS v2 scoring						x	67	25	8	3
	scoring systems										
115	a. Subjective (Likert) scoring	x								100	3
116	b. PI-RADS v2 scoring		x					8	25	67	3

		Agreement		Uncertainty		Disagreement		% Score Distribution of panelists			Abstention
		Consensus	No consensus	Consensus	No consensus	Consensus	No consensus	Score range of **1-3**	Score range **4-6**	Score range **7-9**	n out of 15
	44. The following should be scored on a 1-5 scale for likelihood of involvement?										
117	a. Extracapsular extension (UK Consensus 2013, PI-RADS v2, PI-RADS v1)	x						9		91	4
118	b. SV involvement (UK Consensus 2013, PI-RADS v2, PI-RADS v1)	x						9		91	4
119	c. Bladder neck involvement (UK Consensus 2013, PI-RADS v2)	x						9		91	4
120	d. Neurovascular bundle involvement (UK Consensus 2013, PI-RADS v2)	x						18		82	4
121	e. External sphincter involvement (UK Consensus 2013, PI-RADS v2, PI-RADS v1)	x						9		91	4
122	f. Rectal wall involvement (UK Consensus 2013, PI-RADS v2)	x						9		91	4
123	g. Bladder wall involvement (UK Consensus 2013, PI-RADS v2)	x						9		91	4
124	h. Bone metastases						x	64	27	9	4
	45. To ensure consistency, tumour should be measured as:										
125	a. Maximum single diameter (PI-RADS v2; UK Consensus 2013 only if volume estimation is not possible)						x	69	23	8	2
126	b. 2 axial orthogonal diameters				x			46	46	8	2
127	c. 3-diameters or volume estimation by the product of 3 diameters x 0.52 (UK Consensus 2013)	x						8	8	84	2
128	d. The measured volume by planimetry (contouring of planar areas on each slice of the tumour summated to calculate volume/software rendering, etc)				x			8	42	50	3

		Agreement		Uncertainty		Disagreement		% Score Distribution of panelists			Abstention
		Consensus	No consensus	Consensus	No consensus	Consensus	No consensus	Score range of **1-3**	Score range **4-6**	Score range **7-9**	n out of 15
	46. Peripheral zone (PZ) tumour should be measured from the following sequences										
129	a. T2 only (for TZ in PI-RADS v2)					x		82		18	4
130	b. ADC only (for PZ in PI-RADS v2)					x		82		18	4
131	c. DCE (T1) only					x		100			4
132	d. Any sequence on which it is best seen	x								100	4
	47. Transition zone (TZ) tumour should be measured from the following sequences										
133	a. T2 only (for TZ in PI-RADS v2)				x			9	55	36	4
134	b. ADC only (for PZ in PI-RADS v2)					x		73	18	9	4
135	c. DCE (T1) only					x		73	18	9	4
136	d. Any sequence on which it is best seen	x								100	4
	48. Entire gland volume should be measured on T2-weighted imaging using										
137	a. 3-diameters x 0.52	x								100	3
138	b. Optional volume measurement by planimetry (summation of gland-contoured areas)		x					25	8	67	3
139	c. Mandatory volume measurement by planimetry (summation of gland-contoured areas)					x		100			3

		Agreement		Uncertainty		Disagreement		% Score Distribution of panelists			Abstention
		Consensus	No consensus	Consensus	No consensus	Consensus	No consensus	Score range of **1-3**	Score range **4-6**	Score range **7-9**	n out of 15
	49. The following quantitative metrics should be included within an mpMRI report										
140	a. Prostate gland volume (UK Consensus 2013, PI-RADS v2)	x							8	92	2
141	b. Tumour size (UK Consensus 2013, PI-RADS v2)	x							8	92	2
142	c. Tumour ADC					x		77	23		2
143	d. DCE curve shapes (PI-RADS v1, 2012)					x		85	15		2
144	e. DCE quantitative measures (Time to peak, ktrans, max enhancement, …)					x		92	8		2
	50. On double-reading mpMRI scans										
145	a. All mpMRI should be double-read					x		92	8		3
146	b. Negative scans (scores ≤2) should be double-read if avoiding biopsy is being considered.						x	58	17	25	3
147	c. Equivocal prostate mpMRI scored 3 should be double-read, if avoiding biopsy is being considered.	x						8		92	3
148	d. Positive Scans with a score of 4 or 5 should be double-read before biopsy.					x		92	8		3
149	e. Discordant mpMRI scores with biopsy results should be double-read (retrospectively).	x							16	84	3
	51. Double-reading of mpMRI scans should only be carried out by										
150	a. A bank of tertiary-centre expert radiologists						x	67	33		3
151	b. Experienced radiologists										

		Agreement		Uncertainty		Disagreement		% Score Distribution of panelists			Abstention
		Consensus	No consensus	Consensus	No consensus	Consensus	No consensus	Score range of **1-3**	Score range **4-6**	Score range **7-9**	n out of 15
	52. The structure of a prostate mpMRI report should include:										
152	a. Mandatory narrative report		x					9	27	64	4
153	b. Mandatory pictorial report						x	54	10	36	4
154	c. Mandatory scoring grid					x		82	18		4
155	d. Mandatory snapshots of MRI lesions				x			36	46	18	5
156	53. If pictorial reports are produced, all the sectors within the pictorial report should be scored.						x	58	17	25	3
	54. The pictorial report may be represented on a										
157	a. 12-sector diagram				x			33	17	50	3
158	b. 16-sector diagram (UK Consensus 2013 example)					x		75	8	17	3
159	c. 27-sector diagram (EU Consensus 2011 example)					x		83	17		3
160	d. 36-sector diagram (PI-RADS v2 example)				x			50	17	33	3
	55. In the pictorial report, the prostate diagram should be represented in the following planes:										
161	a. Axial only				x			25	33	42	3
162	b. Axial AND coronal/sagittal				x			42	58		3
163	c. All three planes (UK Consensus 2013, PI-RADS v2)		x					25	17	58	3

No.	Statement	Agreement: Consensus	Agreement: No consensus	Uncertainty: Consensus	Uncertainty: No consensus	Disagreement: Consensus	Disagreement: No consensus	% Score Distribution of panelists: Score range of **1-3**	Score range **4-6**	Score range **7-9**	Abstention: n out of 15
	56. The following number of lesions should be individually drawn within a pictorial report										
164	a. Only the index lesion					x		100			3
165	b. Up to 3 suspicious lesions (past UK Consensus 2013)				x			41	17	42	3
166	c. Up to 4 suspicious lesions (PI-RADS v2)						x	50	17	33	3
167	d. All visible lesions	x						9		91	4
	57. JPEG images or saved key images/snapshots of suspicious lesions should be attached to pictorial reports to illustrate tumour location										
168	as a mandatory requirement					x		84	8	8	3
	58. The following number of suspicious lesions should be attached as JPEG images to the pictorial report or saved as key images in PACS:										
169	a. Only the index lesion					x		82		18	4
170	b. Up to 3 suspicious lesions (past UK Consensus 2013)						x	54	19	27	4
171	c. Up to 4 suspicious lesions (PI-RADS v2)						x	64	18	18	4
172	d. All visible lesions	x						27		73	4
	59. mpMRI suspicious lesions contouring should be performed										
173	a. Only when targeted biopsy or focal treatment is planned.		x					9	36	55	4
174	b. Routinely					x		91	9		4

#	Item	Agreement: Consensus	Agreement: No consensus	Uncertainty: Consensus	Uncertainty: No consensus	Disagreement: Consensus	Disagreement: No consensus	% Score Distribution of panelists: Score range of **1-3**	Score range **4-6**	Score range **7-9**	Abstention: n out of 15
	on the following sequences										
175	a. Anatomical T1W images only					x		100			5
176	b. Anatomical T2W images only		x					18	18	64	4
177	c. T2 and DWI/DCE					x		73	18	9	4
178	d. The sequence it is best depicted on						x	64		36	4
179	e. The sequence required by targeted biopsy fusion software	x						25		75	3
180	[illegible] tumour, (i.e the ''hot-spot'') should be additionally indicated (e.g by contouring, via arrow-heads, etc).	x						18		82	4
	62. Pictorial reporting should be standardised on a										
181	a. Local level		x					14	33	53	0
182	b. Regional level			x				27	27	46	0
183	c. National level		x					13	27	60	0
	63. The format of pictorial reports should be										
184	a. Hand-drawn on paper						x	58	25	17	3
185	b. Generated by computer-assisted tools			x				50	25	25	3
186	c. Either of the above	x						8		92	3

		Agreement		Uncertainty		Disagreement		% Score Distribution of panelists			Abstention
		Consensus	No consensus	Consensus	No consensus	Consensus	No consensus	Score range of **1-3**	Score range **4-6**	Score range **7-9**	n out of 15
	**SECTION IV – QA/QC of mpMRI**										
	**A. SITE-SPECIFIC**										
	64. Sites performing mpMRI should perform scans in adherence with following publications										
187	a. Latest version of PI-RADS						x	57	7	36	1
188	b. Latest UK Consensus guidelines	x							7	93	0
189	c. ESUR Guidelines, PI-RADS v1 2012					x		87	13		0
190	d. European Consensus Recommendations 2011					x		80	13	7	0
191	65. Accreditation for sites offering mpMRI should be set up.		x						33	67	0
	66. Accreditation should be offered by										
192	a. A national body (e.g RCR/SCoR/ISAS/BSUR/BAUS)	x							27	73	0
193	b. A regional body (e.g London Cancer Group, Van guards, Cancer collaboratives)						x	50	33	17	0
194	c. The local trust						x	50	33	17	0
195	d. Private specialised firms					x		73	20	7	0
	performing mpMRI should be										
196	a. 50-100					x		72	14	14	1
197	b. 100-150					x		79	21		1
198	c. 150-250				x			43	53	43	1
199	d. 250-500						x	50	14	36	1
200	e. >500						x	64	15	21	1
201	f. A minimum number is not needed				x			37	18	45	4

		Agreement		Uncertainty		Disagreement		% Score Distribution of panelists			Abstention
		Consensus	No consensus	Consensus	No consensus	Consensus	No consensus	Score range of **1-3**	Score range **4-6**	Score range **7-9**	n out of 15
	68. An accredited centre must										
202	a. Perform mpMRI scanning with T2 and diffusion-weighted imaging (DWI)	x						7		93	0
203	b. Perform mpMRI scanning with T2, DWI and DCE	x						7	7	86	0
	69. Accreditation should be reviewed/renewed/re-assessed on a										
204	a. Every year						x	67	13	20	0
205	b. Every 2 years						x	53	27	20	0
206	c. Every 3 years				x			20	33	47	0
207	d. Every 5 years				x			40	27	33	0
208	e. Never					x		93	7		1
	**B. SCANNER-SPECIFIC**										
209	perform prostate mpMRI.	x								100	0
	71. Quality Assurance of the scanner should be administered by										
210	a. A national body (e.g RCR/SCoR/ISAS/BAUS)				x			43	14	43	1
211	b. A regional body (e.g London Cancer Group, Van guards, cancer collaboratives)					x		86	7	7	1
212	c. The local trust		x					29	7	64	1
213	d. Private specialised firms						x	71	22	7	1
	72. Quality Control should be carried out by one or more of the following										
214	a. An experienced MRI physicist/clinical scientist		x					21	15	64	1
214	b. An experienced radiographer	x						7	7	86	1
214	c. An experienced uro-radiologist	x						7	7	86	1

	Agreement		Uncertainty		Disagreement		% Score Distribution of panelists			Abstention
	Consensus	No consensus	Consensus	No consensus	Consensus	No consensus	Score range of **1-3**	Score range **4-6**	Score range **7-9**	n out of 15
214 73. As minimum scanner QC checks, the automated manufacturer's QC procedure is acceptable.				x			10	60	30	5
74. The following QC metrics should be subjected to assessment (e.g as part of a QC chart system)										
215 a. Gradient calibration	x							22	78	6
216 b. Coil uniformity	x							11	89	6
217 c. Slice thickness	x							22	78	6
218 d. Slice position	x							22	78	6
219 e. Image resolution assessment	x							22	78	6
220 f. Image distortion assessment	x							22	78	6
221 g. Signal to Noise ratio	x							11	89	6
**C. IMAGE-SPECIFIC**										
75. Image quality audits should be documented for										
222 a. Every scan					x		91	9		4
223 b. Every 10 scans					x		73	27		4
224 c. Every 25 scans						x	54	19	27	4
225 d. Randomly selected (10%) proportion of studies	x							16	84	3
76. The level of an acceptable/diagnostic image quality scan should be assessed										
226 a. Qualitatively: by visual methods (e.g assessment of artefacts)	x							25	75	3
227 b. Quantitatively (e.g Signal-to-Noise ratio and Contrast-to-Noise ratio measurements)				x			17	33	50	3

#	Item	Agreement: Consensus	Agreement: No consensus	Uncertainty: Consensus	Uncertainty: No consensus	Disagreement: Consensus	Disagreement: No consensus	% Score Distribution of panelists: Score range of **1-3**	Score range **4-6**	Score range **7-9**	Abstention: n out of 15
	77. Visual image quality should be assessed by an experienced										
228	a. Uro-radiologist giving an opinion on image quality	x						7		93	2
229	b. Radiographer giving an opinion on image quality		x					8	31	61	2
230	c. MRI physicist/scientist giving an opinion on image quality				x			33	50	17	3
231	d. At least two of the above together				x			33	17	50	3
	78. Visual image quality should be assessed on										
232	a. A 1-5 point scale based on personal appreciation of images		x					25	17	42	3
233	b. Against a standard set of images to be used as reference				x			41	42	17	3
	79. Quantitative-based measurements of image quality require										
234	a. Absolute measurements of signal to noise ratio				x			n/a	n/a	n/a	9
235	b. Regular measurements of signal to noise ratio over time (longitudinal assessment to evaluate drifting values over time)	x						n/a	n/a	n/a	9
236	c. Absolute contrast to noise ratio				x			n/a	n/a	n/a	9
237	d. Longitudinal assessment of Contrast to noise ratio over time				x			n/a	n/a	n/a	9
238	80. Reference standards for optimal mpMRI protocols across different scanning platforms should be set up.	x								100	3
	81. Reference standard images should be kept for										
239	a. Local use		x					14	29	57	1
240	b. Regional use				x			21	36	43	1
241	c. National use		x						7	93	1

		Agreement		Uncertainty		Disagreement		% Score Distribution of panelists			Abstention
		Consensus	No consensus	Consensus	No consensus	Consensus	No consensus	Score range of **1-3**	Score range **4-6**	Score range **7-9**	n out of 15
242	82. Quantitative QA assessments of ADC measurements are necessary					x		90	10		5
	83. Significant variations in SNR from the mean over time should be reported and prompt										
243	a. Acquisition protocol checks	x							12	88	7
244	b. Coil checks for defects	x							12	88	7
245	c. Bandwidth checks	x							12	88	7
246	d. More frequent QC measurements				x				50	50	7
	84. The permissible variations in SNR over a year should be										
247	a. 1-5%				x			n/a	n/a	n/a	8
248	b. 5-10%				x			n/a	n/a	n/a	8
249	c. 10-15%				x			n/a	n/a	n/a	8
250	d. 15-20%						x	n/a	n/a	n/a	8
	**D. RADIOLOGIST-SPECIFIC QA**										
251	85. As prostate cancer detection is not part of a national screening programme, minimum achievable QA standards should not be as strict as in breast imaging.						x	54	15	31	2
	86. To ensure that radiologists deliver consistently accurate results over time										
252	a. Self-performance assessment tools should be used	x							21	79	1
253	b. Institution-based audits should be carried out	x								100	1
254	c. External performance assessments should be carried out	x							29	71	1
255	d. Combination of the above	x							7	93	1

		Agreement		Uncertainty		Disagreement		% Score Distribution of panelists			Abstention
		Consensus	No consensus	Consensus	No consensus	Consensus	No consensus	Score range of **1-3**	Score range **4-6**	Score range **7-9**	n out of 15
256	87. External performance assessments should be documented.	x							21	71	1
	88. If an online tool for radiologist's performance assessment is developed, it should be able to										
257	reporting prostate mpMRI for significant cancer.	x							7	93	1
258	b. Compare the radiologists's performance to the mean performance of specialist centres.	x							7	93	1
259	c. Compare the radiologists's performance to the minimum performance level required.	x								100	1
260	d. Evaluate the radiologist's cancer detection rate for each of the mpMRI score		x					14	22	64	1
261	e. Compare the radiologist's cancer detection rate to their local/regional/national peers		x						43	57	1
262	f. Compare the radiologist's cancer detection to expert radiologists.	x								100	1
263	g. Compare the cancer detection rate of radiologists to the prevalence of prostate cancer.										
264	h. Combination of the above	x						7	7	86	1
	89. The radiologist's performance should be assessed										
265	a. Once					x		100			1
266	b. Every 6 months					x		100			1
267	c. Every year						x	64	15	21	1
268	d. Every 2 years				x			28	29	43	1
269	e. Every 3 years		x					29	14	57	1
270	f. Every 5 years				x			50	29	21	1

		Agreement		Uncertainty		Disagreement		% Score Distribution of panelists			Abstention
		Consensus	No consensus	Consensus	No consensus	Consensus	No consensus	Score range of **1-3**	Score range **4-6**	Score range **7-9**	n out of 15
	90. The minimum proportion of mpMRI reports which should be included in the Radiologist's QA audits is likely to be										
271	a. 1%						x	67	33		3
272	b. 5%				x			33	42	25	3
273	c. 10%		x					8	25	67	3
274	d. 20%						x	67	25	8	3
275	e. 25%					x		84	8	8	3
	(after excluding equipment/scanner QA faults)										
276	a. Double-reading of his reports by expert radiologists with sent feedback		x						38	62	2
277	b. Increase frequency of peer-reviewed cases		x						38	62	2
278	c. Increase frequency of MDT attendance	x							16	84	3
279	evaluate	x							23	77	2
280	e. Retraining in expert centres over 3-6 months				x				54	46	2
281	f. Stop reporting					x		70	15	15	2

SECTION V – Management of patients

		Agreement		Uncertainty		Disagreement		% Score Distribution of panelists			Abstention
		Consensus	No consensus	Consensus	No consensus	Consensus	No consensus	Score range of **1-3**	Score range **4-6**	Score range **7-9**	n out of 15
	management options:										
282	a. Age	x								100	2
283	b. Family history	x								100	2
284	c. DRE						x	69	16	15	2
285	d. 5a-reductase inhibitors (finasteride) use	x						8	15	77	2
286	e. Comorbidities	x								100	2
287	f. PSA	x								100	2
288	g. PSA history/kinetics	x							7	93	2
289	h. PSA density	x								100	2
290	Health Index (phi), Stockholm3)				x			46	46	8	2
291	j. Urine test to rule out infection	x						8	8	84	2
292	k. Risk calculator results (PCPT/European calculator/Sherbrook canadian calculator)						x	70	15	15	2
293	l. Any previous biopsy results	x						7		93	2
294	m. Patient’s preference	x							8	92	3
295	n. None of the above (management decisions may be taken on prebiopsy mpMRI scores only)					x		92	8		2
	93. When calculating PSA density,										
296	a. MR-based volume calculated by 3 diameters x 0.52 could be used.	x								100	3
297	b. MR-based volume generated by volumetric software analysis - could be used.	x						8	17	75	3
298	c. TRUS-based volume could be used.					x		85	15		2

	Agreement		Uncertainty		Disagreement		% Score Distribution of panelists			Abstention
	Consensus	No consensus	Consensus	No consensus	Consensus	No consensus	Score range of **1-3**	Score range **4-6**	Score range **7-9**	n out of 15
94. The following management options would be recommended in men with negative mpMRI (scores 1-2)										
a. Discharge					x		92	8		3
b. Discharge based on PSA density below an agreed threshold	x						17	8	75	3
c. Discharge with PSA follow-up to GP if PSA density is below an agreed threshold	x						8		92	3
d. Discharge with PSA follow-up to urologists if PSA density is below an agreed threshold						x	67		33	3
e. Repeat mpMRI if PSA density is above an agreed threshold after a given time interval						x	67		33	3
f. Repeat mpMRI if the man has a family history						x	64	18	18	4
g. Repeat mpMRI if the patient is of an age below an agreed threshold						x	67		33	3
h. Biopsy if PSA density is above an agreed level	x						8	8	84	3
i. Biopsy if there is positive family history				x			33	33	33	3
j. Biopsy if the patient is of an age below an agreed threshold				x			41	42	17	3
k. Biopsy depending on other factors (e.g 5-kallikrein, Prostate Cancer Gene 3 (pca3) (urinary), Prostate Health Index (phi), Stockholm3…)				x			50	33	17	3
l. Biopsy with any biopsy technique						x	50	33	17	3
m. Biopsy with standard transrectal biopsy technique						x	50	42	8	3
n. Biopsy with Transperineal template mapping biopsy technique				x			67	25	42	3
o. Biopsy with any transperineal systematic biopsy technique	x							16	84	3

		Agreement		Uncertainty		Disagreement		% Score Distribution of panelists			Abstention
		Consensus	No consensus	Consensus	No consensus	Consensus	No consensus	Score range of **1-3**	Score range **4-6**	Score range **7-9**	n out of 15
	95. The following management options should be considered in men with equivocal mpMRI (score 3)										
314	a. Discharge					x		100			4
315	b. Discharge if PSA density is below an agreed threshold				x			50	10	40	5
316	c. Discharge with PSA follow-up to GP if PSA density is below an agreed threshold					x					3
317	d. Discharge with PSA follow-up to urologists if PSA density is below an agreed threshold						x	50	25	25	3
318	e. Repeat mpMRI if PSA density is above an agreed threshold						x	58		42	3
319	f. Repeat mpMRI if positive family history						x	58	9	33	3
320	g. Repeat mpMRI if age of patient is below a certain level				x			41	17	42	3
321	h. Biopsy if PSA density is above an agreed threshold	x								100	3
322	i. Biopsy if positive family history		x						33	67	3
323	j. Biopsy if age is below a certain threshold		x					17	25	58	3
324	k. Biopsy depending on other factors (e.g pca 3, …)				x			42	25	33	3
325	l. Biopsy with any biopsy technique						x	50	25	25	3
326	m. Biopsy with standard transrectal biopsy technique						x	50	33	17	3
327	n. Biopsy with any MR-guided targeted biopsy technique		x					17	17	66	3
328	o. Biopsy using transperineal template mapping biopsy technique				x			33	33	33	3
329	p. Biopsy with any transperineal systematic biopsy technique		x					8	33	59	3

		Agreement		Uncertainty		Disagreement		% Score Distribution of panelists			Abstention
		Consensus	No consensus	Consensus	No consensus	Consensus	No consensus	Score range of **1-3**	Score range **4-6**	Score range **7-9**	n out of 15
	96. In men with positive mpMRI (scores 4-5) and negative MR-guided targeted biopsy										
330	Discussion at MDT meeting should be considered	x								100	2
331	97. When performing a targeted biopsy for a lesion scored 3 demonstrated on mpMRI, sampling (systematic biopsy) of the entire gland is recommended.		x					25	17	58	3
332	98. When performing a targeted biopsy for a lesion scored 4-5 demonstrated on mpMRI, sampling (systematic biopsy) of the entire gland is recommended.				x			33	33	33	3
	99. When the mpMRI scores ≥3 and biopsy is negative for significant cancer but shows alternative explanations for the score (eg evidence of atrophy/inflammation/high grade prostatic intraepithelial neoplasia,…), the following should be recommended										
333	a. Discharged				x			25	42	33	3
334	b. Followed-up clinically				x				42	58	3
335	c. Followed-up with repeat mpMRI			x				17	75	8	3

		Agreement		Uncertainty		Disagreement		% Score Distribution of panelists			Abstention
		Consensus	No consensus	Consensus	No consensus	Consensus	No consensus	Score range of **1-3**	Score range **4-6**	Score range **7-9**	n out of 15
	**SECTION VI – Training**										
336	100. Prostate mpMRI reporting can be self-taught.					x		85	15		2
337	101. There should be a competency exam in prostate mpMRI prior to start independent reporting.	x						7	21	72	1
338	102. The attendance to a training course should be made mandatory prior to start independent reporting.	x							14	86	1
339	103. There should be a national accreditation for prostate mpMRI reporting	x							21	79	1
340	breast reporting.						x	50	36	14	1
341	105. There should be evidence of self-directed learning.	x							7	93	1
342	106. Prostate mpMRI training course for non-reporters should be different from the reporters' course and adapted to their specialty field.	x							7	93	1
	107. Certified, standardised Training for Prostate mpMRI should be provided by										
343	a. A national recognised training body (Royal Colleges)	x							7	93	1
344	b. A national society (BSUR, BIR...)	x							14	86	1
345	c. Regional trusts (London Cancer Group, Van guards, Cancer Collaboratives)					x		72	21	7	1
346	d. Local trusts					x		93	7		1

		Agreement		Uncertainty		Disagreement		% Score Distribution of panelists			Abstention
		Consensus	No consensus	Consensus	No consensus	Consensus	No consensus	Score range of **1-3**	Score range **4-6**	Score range **7-9**	n out of 15
	108. Prior to commencing independent mpMRI reporting, reporters should attend at a minimum:										
347	a. A core theoretical mpMRI course	x							7	93	1
348	b. Hands-on practice at workstations	x							7	93	1
	c. Supervised reporting of										
349	i. 50				x			39	38	23	2
350	ii. 100		x					23	23	54	2
351	iii. 150				x			46	8	46	2
352	iv. 200				x			46	23	31	2
	number of										
353	i. 25		x					25	8	67	3
354	ii. 50				x			41	17	42	3
355	iii. 100						x	58	25	17	3
356	iv. 150					x		83	17		3
357	v. 200					x		92	8		3
358	e. Combination of the above	x								100	3
	109. Hands-on training may be given by centres carrying out a minimum number of										
359	a. 50-100 cases/year					x		85	15		2
360	b. 100-150 cases/year						x	69	31		2
361	c. 150-250 cases/year				x			39	15	46	2
362	d. ≥ 250 cases/year	x							7	93	2
